# T‐cell adoptive immunotherapy for BK nephropathy in renal transplantation

**DOI:** 10.1111/tid.13399

**Published:** 2020-07-14

**Authors:** Sadia Jahan, Carla Scuderi, Leo Francis, Michelle A Neller, Sweera Rehan, Pauline Crooks, George R Ambalathingal, Corey Smith, Rajiv Khanna, George T. John

**Affiliations:** ^1^ Kidney Health Service Royal Brisbane and Women's Hospital Herston Qld Australia; ^2^ Pathology Department Royal Brisbane and Women's Hospital Herston Qld Australia; ^3^ QIMR Berghofer Centre for Immunotherapy and Vaccine Development QIMR Berghofer Medical Research Institute Herston Qld Australia

**Keywords:** BK virus, BKPyV nephropathy, T‐cell adoptive immunotherapy

## Abstract

**Introduction:**

BK virus (BKPyV) nephropathy occurs in 1%‐10% of kidney transplant recipients, with suboptimal therapeutic options.

**Case:**

A 54‐year‐old woman received a transplant in March 2017. BKPyV was detected at 1.5 × 10^2^ copies/mL within a month, necessitating halving of mycophenolate and addition of leflunomide. Allograft histology in December showed polyomavirus nephropathy treated with intravenous immunoglobulin and cessation of mycophenolate. In February 2018, cidofovir and ciprofloxacin were commenced. In April, tacrolimus was reduced while introducing everolimus. A second graft biopsy in August showed increasing polyoma virus infection and a subsequent biopsy in September for worsening renal function showed 30% of tubular reactivity for simian virus 40 (SV40). Allogeneic BKPyV‐reactive T cells were generated from the patient's daughter and infused over 10 sessions starting late September. The fourth allograft biopsy in November 2018 demonstrated involvement of BKPyV in 50% of tubules. Allograft function continued to decline, requiring hemodialysis from December 2018. Allograft nephrectomy after 6 months showed <1% SV40 in preserved tubules and 80% interstitial fibrosis.

**Discussion:**

We conclude that the T‐cell adoptive immunotherapy reduced BKPyV load significantly despite extensive infection, but attendant fibrosis and tubular atrophy led to graft failure. Early intervention with T‐cell therapy may prove efficacious in BKPyV nephropathy.

## 1 INTRODUCTION

BK virus (BKPyV) nephropathy occurs in 1%‐10% of kidney transplant recipients (KTR).^1^ The therapeutic options are suboptimal for eradication and cure.^2^

We describe BKPyV nephropathy (BKPyVAN) in a KTR who was treated unsuccessfully with reduction in immunosuppression, leflunomide, intravenous immunoglobulin, cidofovir, and ciprofloxacin. Finally, she received T‐cell adoptive immunotherapy that achieved a reduction in BKPyV (<1%) in renal tubules, but the allograft was lost to interstitial fibrosis and tubular atrophy (IFTA).

## 2 CASE PRESENTATION

A 54‐year‐old woman received a deceased donor kidney transplant in March 2017 for end‐stage kidney disease from diabetic nephropathy. She was unsensitized and received a five‐HLA‐mismatched kidney with an uneventful post‐operative course. She was treated with Basiliximab® induction and maintained on tacrolimus, prednisolone, and mycophenolate.

BKPyV was detected at 1.5 × 10^2^ copies/mL within a month of transplantation leading to halving of mycophenolate. The BKPyV count increased to 1.7 × 10^4^/mL. Leflunomide therapy was started and maintained targeting a teriflunomide level of 50 mg/L. Allograft histology in December 2017 showed polyomavirus nephropathy, with nuclear reactivity for simian virus 40 (SV40) large T antigen in 3% of tubules. There was no interstitial fibrosis in this biopsy. Intravenous immunoglobulin was infused at weekly intervals over 10 sessions and mycophenolate stopped. In February 2018, cidofovir 20 mg was infused at fortnightly intervals for eight doses and ciprofloxacin 750 mg twice daily was commenced. In April, tacrolimus was reduced to achieve a trough of 3‐4 µg/L while introducing everolimus (target trough 2‐3 µg/L).

Blood BKPyV count increased to 4.4 × 10^4^/mL. A second graft biopsy in August 2018 showed polyoma virus in approximately 8% of tubules. There was mild interstitial fibrosis (10%).

A third graft biopsy in September 2018 for worsening renal function showed more extensive reactivity for SV40 (involving 30% of tubules). This biopsy was, however, quite small for accurate assessment of fibrosis.

**Figure 1:**
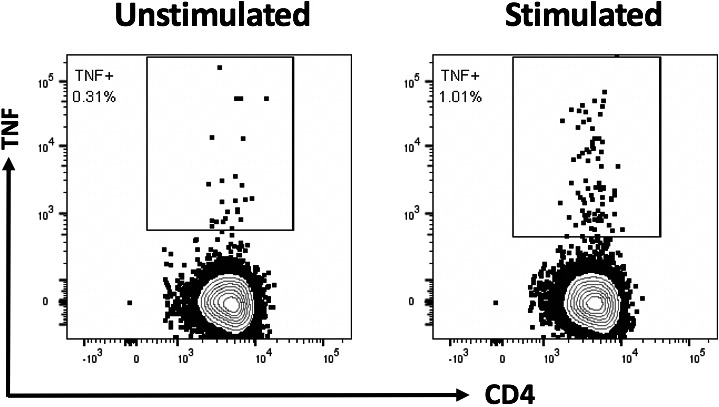
BKPyV‐specific function of the adoptively transferred T cells. Peripheral blood mononuclear cells isolated from the haplo‐matched related donor were stimulated with a pool of BKPyV peptides and cultured in the presence of interleukin 2 for 17 d. The production of tumour necrosis factor (TNF) by the resultant T cells was assessed following re‐stimulation with the BKPyV peptide pool. Representative flow cytometry plots show the CD4^+^ T‐cell response to BKPyV epitopes

Adoptive immunotherapy was then provided under the Special Access Scheme of the Australian Therapeutic Goods Administration. Allogeneic BKPyV‐reactive T cells were generated from the patient's haplo‐matched daughter as previously described in literature, but using peptide epitopes from BKPyV instead of cytomegalovirus.^3^, ^4^ The expanded T cells had a detectable response to BKPyV epitopes; this was evidenced by tumor necrosis factor production upon recall with the pool of BKPyV peptide epitopes used to generate the T‐cell therapy (Figure 1). The frequency of BKPyV‐specific T cells prior to administration of T‐cell product was assessed, but the level of T‐cell response to BKPyV was undetectable.

Intravenous infusions of 3.6 × 10^7^ cells from the patient's daughter were administered weekly over 10 sessions, starting late September 2018. During this period, creatinine continued to increase, and for a possible concomitant rejection, she received three doses of intravenous methylprednisolone; however, rejection was not seen on biopsy. The fourth allograft biopsy in November 2018 demonstrated involvement of BKPyV in 50% of tubules. There was interstitial fibrosis involving 50% of the cortex.

**Figure 2:**
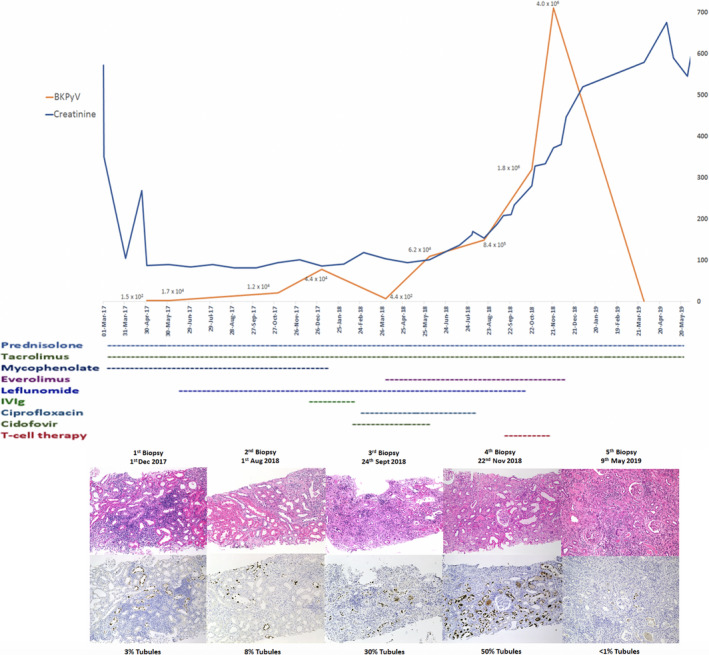
Longitudinal analysis of creatinine levels, BKPyV counts, therapeutic interventions and histology showing proportion of affected tubules

The profile of creatinine, BKPyV count, and allograft histology is plotted against time course and therapy in Figure 2.

Allograft function continued to decline, requiring maintenance hemodialysis from December 2018. An allograft nephrectomy after 6 months showed extensive (approximately 80%) interstitial fibrosis. The tubules in the preserved cortical areas showed only rare immunohistochemical reactivity for SV40 (<1%).

We conclude that the T‐cell adoptive immunotherapy had reduced BKPyV load, but with extensive infection, despite viral clearance, attendant fibrosis and tubular atrophy caused allograft failure.

Our patient has no residual BK viremia 6 months post nephrectomy and is relisted for a second renal transplantation.

## 3 DISCUSSION

Fifty percent of KTRs with BKPyVAN lose allografts.^5^ Reduction of immunosuppression is the first line therapy for transplantation recipients with BK reactivation.^1^ Treatment options for BKPyVAN are leflunomide, intravenous immunoglobulin, ciprofloxacin, cidofovir, and lastly, adoptive T‐cell therapy. These are described in the literature with varying degrees of success. For our patient, all these options were trialed starting with reduction in immunosuppression and commencement of leflunomide. Intravenous immunoglobulin was then trialed without any reduction in BKPyV count. Ciprofloxacin and cidofovir were next trialed with careful monitoring of renal function due to cidofovir's known nephrotoxicity. Although renal function remained stable initially, BKPyV count worsened with histology showing extensive involvement of SV40. Decision was then made to trial T‐cell adoptive immunotherapy.

In adoptive T‐cell therapy, primed BKPyV‐reactive T cells are expanded and infused to control BKPyVAN.^1^ Balduzzi et al reported a patient with hematopoietic stem cell transplantation with progressive multifocal leukoencephalopathy responding to donor‐derived JC virus‐specific T cells.^6^ A similar approach targeting BKPyV homologs in seven patients with reactivated virus after stem cell transplantation achieved complete remission in five and a partial response in one, while one patient lacking activity against BKPyV did not respond.^7^ Lamarche has described a protocol for obtaining third‐party (not recipient or donor) derived BKPyV‐specific T‐cell lines.^8^

Our patient had near‐complete clearance of BKPyV following T‐cell therapy on histology, but the attendant IFTA had resulted in graft failure. After adoptive immunotherapy, there was a reduction in SV40‐positive cells in areas of the allograft free from IFTA, from 50% to < 1% tubular involvement. However, the biopsy immediately after the infusions showed increasing BKPyV presence, suggesting the process indeed occurs slowly. Earlier use of T‐cell therapy, when viral load is lower, may improve the impact of this therapy. Parenchymal scarring with tubular loss, atrophy, and fibrosis remains the sequelae of BKPyVAN even when the infection is cleared after reduction of immunosuppression.^9^, ^10^, ^11^

T‐cell adoptive immunotherapy for BKPyVAN warrants trialing early in the course of illness. Early intervention with T‐cell therapy, perhaps before cidofovir, may prove efficacious and avoid nephrotoxicity.

## CONFLICT OF INTEREST

The authors declare no conflict of interest.

## AUTHOR CONTRIBUTIONS

All authors contributed to the final manuscript. SJ, CS, CS, RK, and GTJ conceived of the idea for presentation. LF carried out examination of histology, MN coordinated regulatory approvals and T‐cell therapy supply, SR manufactured the T‐cell therapy, and GRA and PC performed laboratory studies. All authors provided critical feedback and shape the manuscript to its current form.
